# Perinatal Renal Vein Thrombosis: Role of Imaging in the Initial Diagnosis

**DOI:** 10.5334/jbsr.3263

**Published:** 2023-09-25

**Authors:** Ana Filipa Forjaco Jorge, Pedro Rodriguez Rios Riesenberger, Maria Eugénia Trindade Soares

**Affiliations:** 1 Member of the Belgian Society of Radiology, Hospital Dona Estefânia – Centro Hospitalar Universitário de Lisboa Central, Lisbon, PT; 2 Hospital Dona Estefânia – Centro Hospitalar Universitário de Lisboa Central, Lisbon, PT

**Keywords:** perinatal renal vein thrombosis, neonatal ultrasound, neonatal Doppler study

## Abstract

We report the case of a neonate who presented with a left abdominal flank mass and hemodynamic instability signs, with the final diagnosis of left renal vein thrombosis complicated with inferior vena cava thrombosis and perirenal hematoma. We illustrate the importance of an early imaging approach with ultrasound and Doppler evaluation to finally diagnose perinatal renal vein thrombosis and its complications.

**Teaching Point:**

An early imaging approach with ultrasound and Doppler evaluation is fundamental to finally diagnose perinatal renal vein thrombosis and its complications.

## Introduction

Renal vein thrombosis (RVT) is a rare perinatal event, with an incidence of 2.2 per 100000 live births [1]. Greyscale ultrasound (US) and Doppler evaluation are the gold-standard imaging techniques in neonates with suspected RVT [2].

## Case History

On the fourth day of life, a female term neonate was transferred to our neonatal intensive care unit, presenting with a 7 cm non-pulsatile tender mass in the left abdominal flank and signs of hemodynamic instability. Initial laboratory evaluation showed reduced hemoglobin concentration (8.7 g/dL) and prothrombin time (9 s).

Because an intraabdominal hematoma was strongly suspected, the pediatric radiologist promptly performed an abdominal US and additional Doppler evaluation of the kidneys.

**Figure 1:**
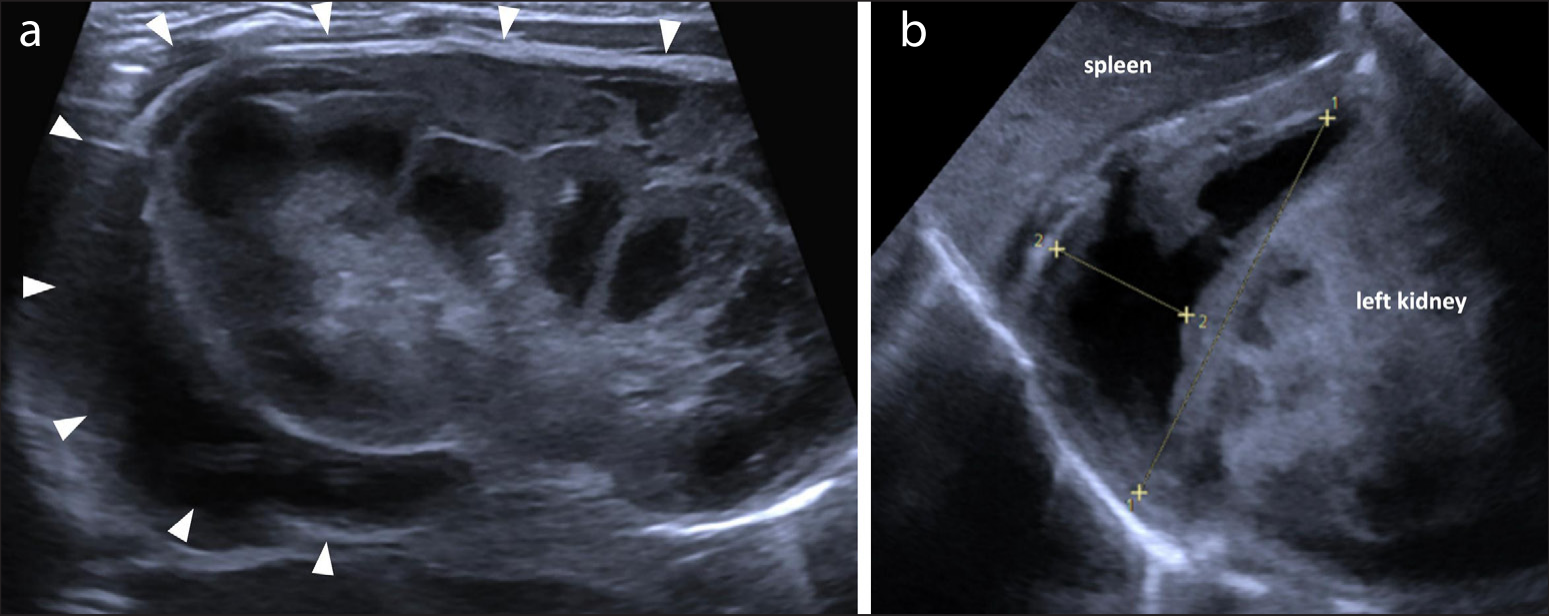
**(a)** US coronal oblique plane on the left flank, demonstrating enlargement and increased cortical echogenicity of the left kidney, with a thick perirenal hematoma (arrowheads). **(b)** US demonstrating an associated left adrenal hematoma, with hypoechoic areas of liquefaction.

**Figure 2:**
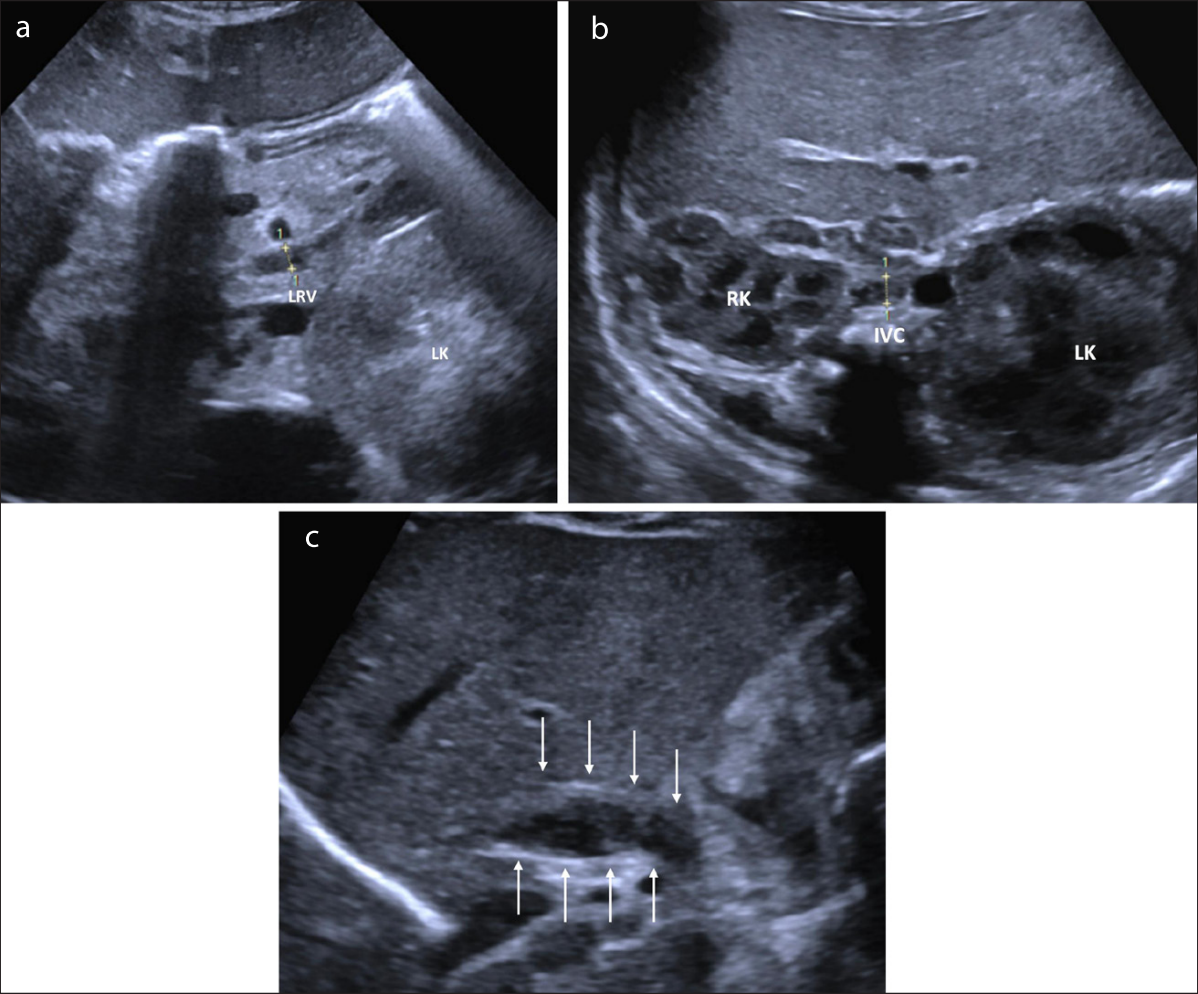
**(a)** US axial plane of the left flank, demonstrating the enlarged left kidney (LK) and left renal vein (LRV), filled with hypoechoic content. **(b)** US axial plane of the abdomen, demonstrating the asymmetric kidneys (RK, LK) and the inferior vena cava (IVC) filled with hypoechoic content. **(c)** US sagittal plane demonstrating the enlarged suprarenal IVC, filled with hypoechoic content (arrows).

**Figure 3:**
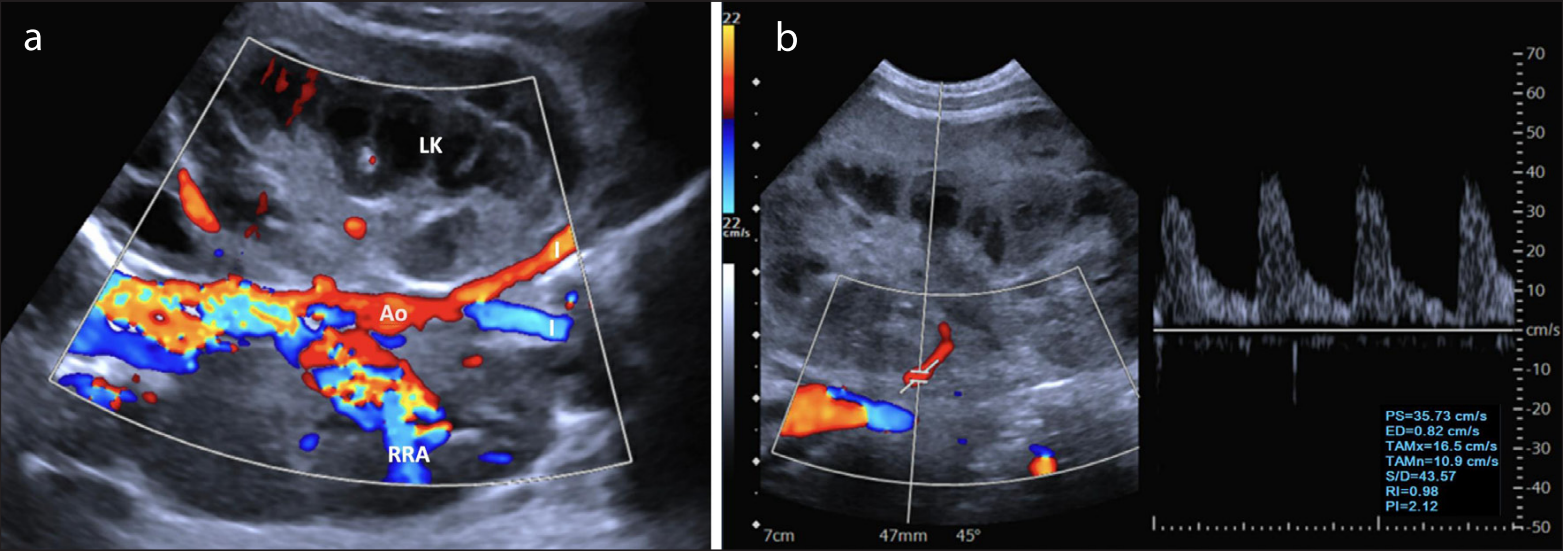
**(a)** Color Doppler on the left kidney (LK) revealing an absence of venous flow in the lesser tributary veins and in the main renal vein, and a reduced flow in the main renal artery, with exuberant arterial flow in the contralateral renal artery (RRA), aorta (Ao) and iliac branches (I). **(b)** Color and spectral Doppler on the left kidney revealing a decreased diastolic flow and increased resistance index (RI) in the renal artery.

US detected an enlarged left kidney, with a 7 cm bipolar length (while the mean expected values in this age are 4.5 cm, with a standard deviation of 0.3 cm [3]). There was also an increased left kidney cortical echogenicity and a thick perirenal hematoma extending continuously to the left adrenal gland area (Figure 1a, 1b). There was an increased caliber of the left renal vein (LRV) and the inferior vena cava (IVC), both filled with hypoechoic content (Figure 2a–2c). Color Doppler imaging revealed an absence of venous flow in the LRV and the lesser tributary veins of the left kidney (Figure 3a), as well as the IVC until the level of the hepatic veins, confirming complete thrombosis. Spectral Doppler of the left renal artery showed a decreased diastolic flow and increased resistance index (Figure 3b). Findings were consistent with left RVT complicated with IVC thrombosis and perirenal and adrenal hematoma.

Investigation for causal factors revealed a homozygotic factor V Leiden mutation.

Subcutaneous anticoagulation therapy with enoxaparin was initiated.

**Figure 4:**
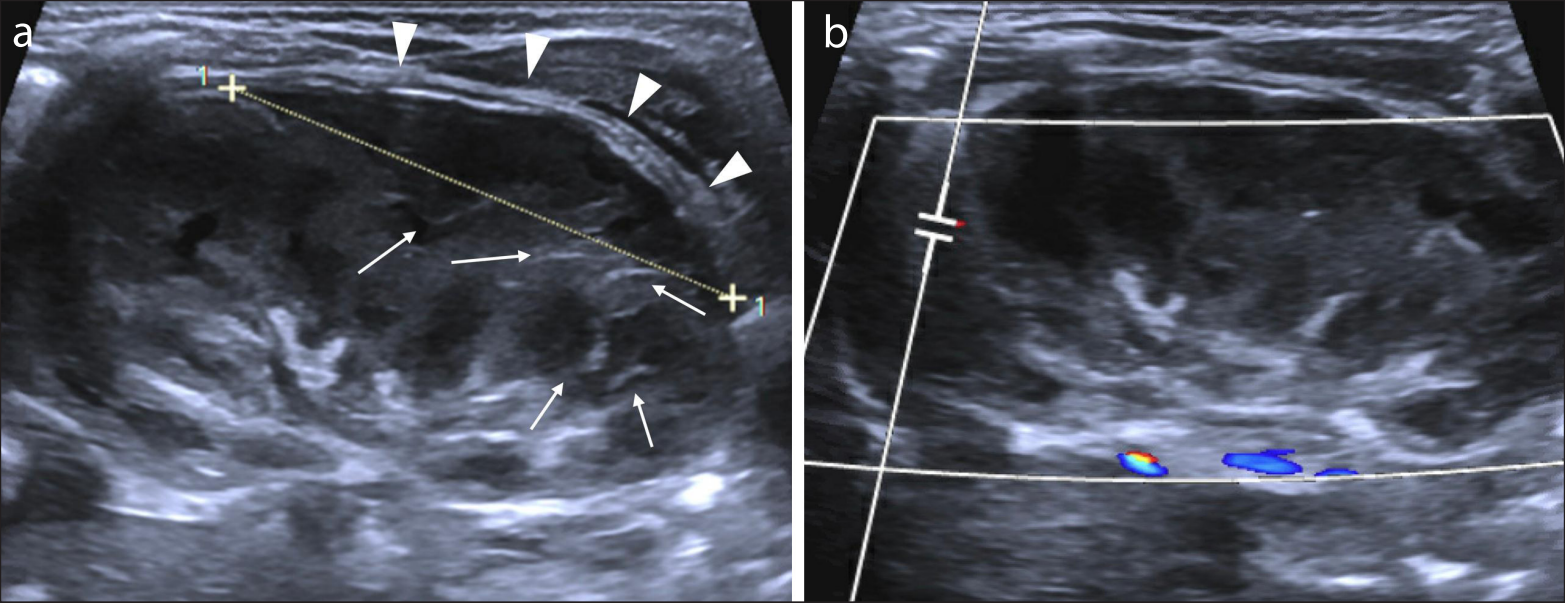
**(a)** Follow-up US two weeks later revealing a reduction in the perirenal hematoma thickness (arrowheads), and a decreased kidney cortico-medullary differentiation, with an intermedullary streaking pattern (arrows). **(b)** Doppler imaging two weeks later demonstrating an almost complete loss of vessel flow in the left kidney.

**Figure 5:**
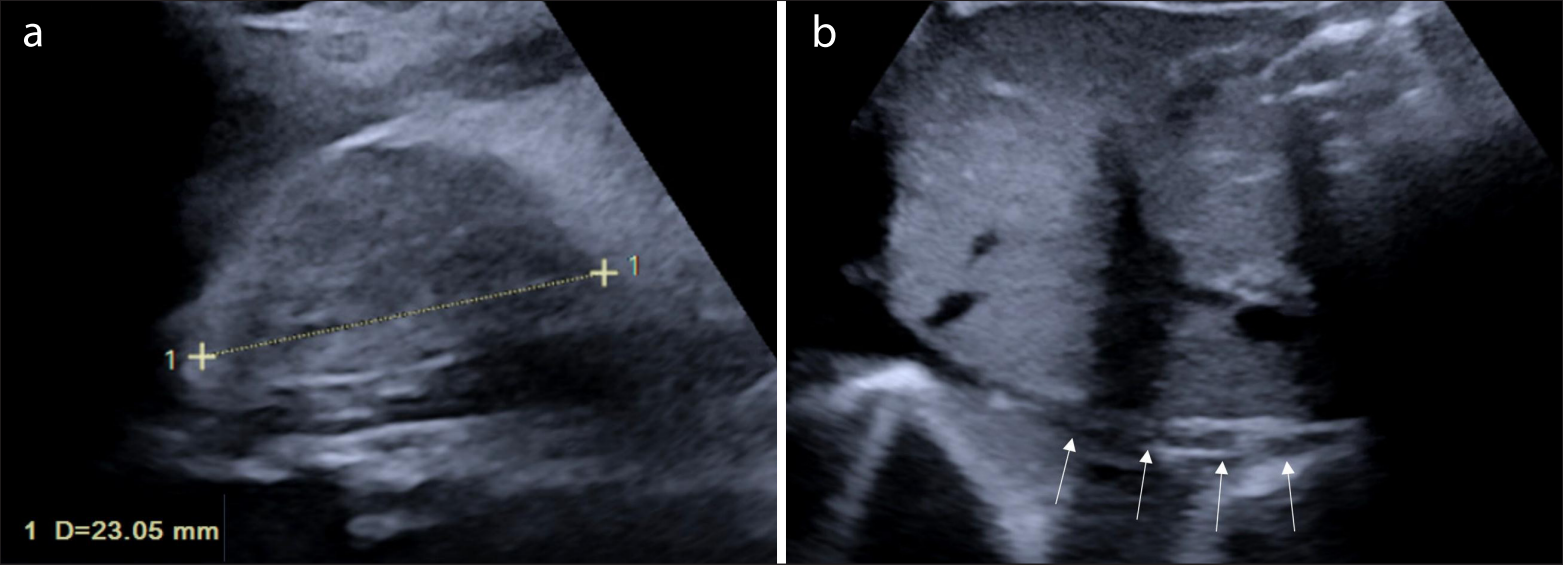
**(a)** US one year later revealing an utterly atrophic left kidney. **(b)** US one year later revealing a filiform hypoechoic IVC (arrows).

Follow-up imaging two weeks later revealed a reduced size of the left kidney, with loss of its cortico-medullary differentiation, and an intermedullary streaking pattern composed of hyperechoic streaks surrounding hypoechoic medullas (Figure 4a). There was persistence of the venous thrombus. The arterial evaluation demonstrated a progressive flow loss in the main renal artery (Figure 4b). One year later, the left kidney was atrophic (Figure 5a), and the IVC was filiform, with no internal flow (Figure 5b).

## Comments

RVT is a rare perinatal complication, in which a thrombus forms in the arcuate and interlobular renal veins, successively extending into the renal vein [2].

Risk factors include inherited thrombophilia, polycythemia, acute perinatal diseases, and maternal thrombotic states. The factor V Leiden mutation has a clear pathogenic role [4].

Clinical presentation includes one of three cardinal signs: palpable flank mass (45%), macroscopic hematuria (55%), and thrombocytopenia (45%) [4].

Baseline laboratory tests must comprise platelet count and prothrombin time [4].

Neonatal US depicts the initial features of RVT, including a generalized edematous renal enlargement with decreased cortico-medullary differentiation or an overall increased cortical echogenicity [2,4]. In the first two weeks, a vascular or perivascular hyperechoic streaking pattern may become evident, owing to the presence of interlobular and interlobar thrombus [2,5]. Patchwork appearances of hyperechoic hemorrhagic areas and hypoechoic edematous zones may also develop [5]. Finally, the kidney may gradually recover its normal appearance, but, eventually, the opposite may also occur, leading to renal atrophy [2,5].

Since the presentation, a hypoechoic thrombus may be seen in the renal vein, extending to the IVC [4]. Doppler imaging confirms the diagnosis, with an absent intrarenal and main renal venous flow, decreased renal arterial diastolic flow and increased arterial resistance indices [6].

Perirenal hematoma is a very rare finding in neonates and may result from capsular distension and consequent rupture by generalized edema and hemorrhage of the kidney [7]. Adrenal hemorrhage occurs in 15% of the cases, by the same mechanism, more frequently on the left, where the left adrenal vein drains into the left renal vein [2].

Extension of thrombosis to the IVC occurs in 45% of the cases, demonstrated on US as the presence of reflective material within the venous lumen, and flow defect on Doppler imaging [2].

Treatment options include supportive therapy, anticoagulation for unilateral RVT with renal function impairment or IVC extension, and thrombolysis for bilateral RVT [4].

RVT is often associated with long-term kidney atrophy, systemic hypertension, and chronic kidney disease [2,4].

## Conclusion

We present this case to highlight the role of imaging in the early detection of perinatal RVT, a rare and potentially fatal complication.
